# Special Issue “Molecular Advances in Heart Disease: Genomics, Proteomics, and Bioinformatics of Heart Research”

**DOI:** 10.3390/ijms262110492

**Published:** 2025-10-29

**Authors:** Justyna Fert-Bober

**Affiliations:** Advanced Clinical Biosystems Research Institute, Smidt Heart Institute, Cedars-Sinai Medical Center, 127 San Vicente Blvd #A9100, Los Angeles, CA 90048, USA; justyna.fert-bober@cshs.org

## 1. Introduction

Cardiovascular diseases (CVDs) remain the leading cause of mortality worldwide and encompass a wide spectrum of conditions, including coronary artery disease, myocardial infarction, heart failure, arrhythmias, valve disease, hypertension, congenital and inherited heart conditions, myocarditis, and fibrosis [1]. The complex interplay among genetic predisposition, molecular alterations, chronic inflammation, extracellular matrix remodeling, and aging underscores the multifactorial nature of heart diseases.

In recent years, remarkable advances in genomics, proteomics, and bioinformatics have transformed our understanding of the pathobiology of CVD. These molecular approaches have enabled comprehensive exploration of genetic variants, transcriptional regulators, protein modifications, noncoding RNAs, and biomarker signatures, collectively shedding light on the mechanisms of disease initiation, progression, and therapeutic response [2].

However, understanding the heterogeneity of cardiovascular disease requires more than cataloging molecular changes; it demands integration. Combining diverse data into large-scale, multi-omics frameworks provides a mechanistic foundation for precision medicine. For instance, network-based approaches such as atrial fibrillation have been applied to delineate endophenotypes [3], while ontology-driven profiling has illuminated the complexity of congenital heart defects [4,5]. Building on these strategies, machine learning and artificial intelligence now offer powerful means to interpret high-dimensional data for biomarker discovery and outcome prediction [6].

Against this backdrop, this Special Issue, ‘Molecular Advances in Heart Disease: Genomics, Proteomics, and Bioinformatics of Heart Research’, brings together original research and reviews that showcase how molecular technologies and computational tools are reshaping cardiovascular science.

## 2. Genomics and Bioinformatics

Within this broader landscape, genomic and bioinformatic approaches provide some of the clearest examples of how large-scale molecular data can be leveraged to dissect cardiovascular disease mechanisms and guide personalized care. In this Special Issue, Bukaeva et al. [7] evaluated the diagnostic yield of genetic testing in patients with long QT syndrome (LQTS), including those with borderline clinical scores. Their findings demonstrate that genomic screening can identify pathogenic variants even in patients with low Schwartz scores, suggesting that additional rare variants in cardiac genes may act as modifiers contributing to disease severity and heterogeneity [7]. Similarly, an illuminating study by Guimarães de Oliveira et al. [8] employed a multi-strategy bioinformatics pipeline to unpack the molecular underpinnings of congenital heart defects (CHDs). They combined gene and phenotype ontology analyses (drawing from Gene Ontology and Human Phenotype Ontology) with systems biology network modeling and differential gene expression across multiple CHD types. Using this ontology-based network and differential gene expression framework, they identified new CHD-associated genes and pathways. The key network players included EP300, CALM3, EGFR, NOTCH1, TNNI3, and SMAD4, while novel expression patterns implicated immune and metabolic processes in CHD pathogenesis. These results align with meta-analyses of Tetralogy of Fallot expression datasets and population-scale sequencing studies [9], which similarly point to chromatin regulators, ciliary proteins, and developmental transcription factors as convergent contributors.

Together, these studies underscore how multi-omics and network-based strategies converge to elucidate the molecular architecture of complex cardiac disorders.

## 3. Transcriptomics and Gene Regulation

If genomics lays the foundation, transcriptomics provides a dynamic view of how gene regulation shapes cardiac adaptation and development [10].

In this Special Issue, several articles highlight how transcriptional regulation governs adaptive and developmental processes. Głogowska-Ligus et al. [11] described a new role of zyxin as a stress-responsive safeguard during acute injury, with its upregulation in acute coronary syndrome and stable coronary artery disease suggesting its role in the activation of cellular repair mechanisms [11]. These findings complement previous findings that zyxin functions as a mechanotransducer to promote cardiomyocyte survival and limit fibrosis under conditions of hemodynamic overload [12]. In a developmental study, M. Rezaul Hasan et al. explored the Forkhead box transcription factor jumu in Drosophila and identified more than 1200 dysregulated genes in jumu-deficient embryos, establishing its role as a master regulator of cardiogenic cell division [13]. Translationally, Inoue et al. examined transcriptomic changes in the skeletal muscle of rats with cardiac cachexia, revealing how aerobic training reshaped gene expression patterns associated with wasting [14]. Together, these findings highlight transcriptomic regulation as a central mechanism in both adaptive repair and developmental control.

## 4. RNA Biomarkers

Bridging basic mechanistic studies and their clinical application, circulating RNA molecules have emerged as powerful diagnostic tools [15]. Research has shown that microRNAs (miRNAs), stable, short, noncoding RNAs, can serve as diagnostic biomarkers and potential therapeutic targets in CVD [16,17,18,19]. In this context, So-Yeon Kim et al. revealed 29 miRNAs that were differentially expressed in the acute myocardial infarction (AMI) group compared to the control group. Notably, he reported that exosomal microRNA-486-5p levels are significantly elevated in AMI and closely correlated with high-grade atherosclerotic plaques [20]. These findings complement those of prior studies showing the regulatory roles of miR-33, miR-27b, miR-148a, and miR-223 in cholesterol transport, lipoprotein metabolism, and atherogenesis [21,22,23,24]. The antagonism of specific miRNAs, such as miR-33, miR-148a, and miR-652-3p, improves lipid handling and expression of the endothelial repair cycline d2 gene (Ccnd2), which reduces atherosclerosis lesion formation, suggesting therapeutic potential [25]. Collectively, these studies underscore the promise of miRNAs as biomarkers and therapeutic targets across cardiovascular conditions.

## 5. Complementary Reviews

Beyond original research, this Issue also features reviews that situate molecular discoveries in a translational and therapeutic context. Highlights include analyses of the role of microRNAs in cardiovascular disease [26], the cardiometabolic impact of sodium–glucose cotransporter 2 inhibitors in aortic stenosis [27], and the use of DNA methylation biomarkers for risk stratification [28]. From genomics and transcriptomics to circulating biomarkers and therapeutic modulation, these thematic approaches illustrate how molecular biology and omics technologies are reshaping our understanding of CVD and informing precision diagnostics and interventions.

## 6. Summary

We hope the insights shared in this Special Issue will spark innovative ideas, inspire collaborations across fields, and help molecular discoveries achieve real-world clinical impact. Looking ahead, one of the most exciting directions for cardiovascular research is bringing together multi-omics, spatial, and longitudinal data to better capture how disease unfolds in real time and across diverse populations. New tools, like single-cell multi-omics, spatial transcriptomics, and deep proteome profiling, are opening the door to understanding how specific cell types contribute to disease and how the heart and vessels are dynamically remodeled.

At the same time, advances in patient-derived organoids, engineered cardiac tissues, and multiorgan systems are creating powerful testbeds for converting genomic and bioinformatic discoveries into mechanistic insight and translational progress. Equally important is making sure that machine learning and AI tools for biomarker discovery and risk prediction are not only powerful, but also transparent, explainable, and validated across multiethnic, long-term cohorts.

Expanding our lens to include sex differences, aging, and gene–environment interactions will further help improve patient stratification and therapeutic strategies. Ultimately, realizing the vision of personalized cardiology will depend on teamwork across disciplines, open data-sharing on a global scale, and weaving together molecular signatures with imaging, clinical phenotyping, and digital health technologies.
